# Paternal involvement and peer competence in young children: the mediating role of playfulness

**DOI:** 10.3389/fpsyg.2025.1477432

**Published:** 2025-03-19

**Authors:** Chunyan Liang, Xinwen Bi

**Affiliations:** Department of Education, Shandong Women’s University, Jinan, Shandong Province, China

**Keywords:** paternal involvement, young children, playfulness, peer competence, Chinese context

## Abstract

**Objective:**

The capacity to interact with peers during early childhood can profoundly and enduringly influence later development and adaptation. Previous research has indicated that paternal involvement plays a vital role in shaping children’s peer competence. However, limited research has been conducted on this association within the Chinese cultural contexts or on the potential mechanisms that underlie it. Therefore, the present study aims to investigate whether there is a close link between paternal involvement and peer competence in Chinese young children, as well as whether children’s playfulness mediates this relationship.

**Method:**

The Chinese version of the Paternal Involvement Questionnaire (FIQ) was distributed to 359 fathers with children (4–6 years old). Children’s Playfulness Scale (CPS) and Ability to Associate With Partners Scale (AAPS) were distributed to the children’s mothers.

**Results:**

(1) There are positive correlations between paternal involvement, young children’s playfulness and peer competence after controlling for the demographic variables of age and gender. (2) Paternal involvement is positively related to young children’s peer competence. (3) Playfulness partially mediated the relationship between paternal involvement and children’s peer competence. Findings from this study emphasize the significance of paternal involvement in enhancing young children’s peer competence, while also highlighting the value of positive emotional traits such as playfulness for fostering family interaction and promoting young child development.

## 1 Introduction

Early childhood is a critical period for children’s social development, during which peers play an irreplaceable role (Shaffer, 2005; Zhang, 1999). Interactions with peers offer a significant developmental context for children to acquire a diverse range of behaviors, skills, and attitudes that profoundly impact their adaptation throughout the lifespan. Previous studies consistently demonstrate that peer competence serves as a protective factor in facilitating children’s overall development (Parker et al., 2015). A high level of peer competence is characterized by reciprocal interactions, cooperative behaviors, intimate relationships, and collaborative problem-solving skills that are positively associated with children’s mental well-being and social adaptation (Eggum-Wilkens et al., 2014; Wang et al., 2020). Given the significant role of peer competence, it is critical to investigate its predictors and explore the underlying mechanisms.

With the development of family theory and positive developmental psychology, researchers have started recognizing the family processes that contribute to social competence, peer acceptance, and the ability to establish and maintain qualitatively enriching friendships (Ladd and Parke, 2021; Parker et al., 2015). Particularly noteworthy are paternal factors on family dynamics and child development (Cabrera, 2020; Flouri et al., 2016). China’s rapid socioeconomic transformation over the past two decades has precipitated significant shifts in family dynamics, with there being growing recognition of the crucial role paternal involvement plays in contemporary child-rearing practices (Li, 2020; Li and Lamb, 2016; Liu et al., 2021). Our study specifically focuses on examining the relationship between positive paternal involvement and children’s peer competence. As an extension, we further investigate the mediating role of children’s playfulness in this relationship within the Chinese context.

### 1.1 Relationship between paternal involvement and children’s peer competence

The family-peer system linkage theory posits that the family is the primary environment for children’s socialization, and that parenting behaviors and patterns have a significant impact on children’s social competence (Parke, 2004). Previous research has focused on the influence of mothers, while the influence of fathers has been understudied (Li, 2020; Zhang, 2013). Existing studies have consistently shown that paternal involvement during early childhood significantly contributes to offspring development in cognitive, emotional, behavioral, and social adaptation domains (Choi and Song, 2014; Liu et al., 2021; Rollè et al., 2019; StGeorge and Freeman, 2017). Children residing with their fathers show better adaptive functions, fewer problem behaviors, and more harmonious interpersonal relationships (Lamb, 2010). Longitudinal studies have further revealed that early paternal participation can serve as a predictor of later peer problems among children (Craig et al., 2018; Flouri et al., 2016).

In addition, evidence suggests that maternal and paternal parenting may have distinct impacts on children’s social development. Neuroscience research indicates that maternal parenting can stimulate children’s affective regions, whereas paternal parenting can stimulate socio-cognitive regions (Abraham and Feldman, 2022; Kopala-Sibley et al., 2020). Developmental psychology research suggests that fathers and mothers influence children’s social development through different mechanisms, such as mother–child attachment relationships (Bowlby, 1969) and father-child activation relationships (Paquette, 2004). According to attachment theory, mothers usually provide a safe haven by calming and soothing children when they are distressed. In contrast, according to activation relationship theory, fathers typically engage in physical play with young children more frequently than mothers, and father-child physical play (e.g., rough and tumble play) provides a secure base for children to safely explore the outside world, learn to cope with unfamiliar situations and challenges, and develop social emotional skills (Amodia-Bidakowska et al., 2020; Paquette et al., 2021; Paquette and StGeorge, 2023).

While these findings imply the unique value of paternal parenting in child development and a robust association between paternal involvement and young children’s peer competence, there remains a dearth of research investigating the underlying mechanisms. In this study, we aim to explore the mediating role of playfulness in the relationship between paternal involvement and children’s peer competence within a Chinese cultural context, thereby elucidating one such mechanism.

### 1.2 Mediating role of playfulness

Play is an innate inclination towards curiosity, imagination, and fantasy, and child-directed play is inherently developmentally appropriate and can be harnessed to facilitate academic and social learning (Wong et al., 2008). Some scholars have recognized the crucial role of play in young children’s cognitive, emotional, and social development (Barnett, 1990; Lieberman, 1977). Prior studies primarily focused on the external manifestation of play and its associations with language and cognition (Reddy, 1991). However, advancements in play theory and positive developmental psychology have prompted researchers to consider playfulness as an active tendency or a personality trait, and study it from clinical (Bögels and Phares, 2008), developmental (Hwang, 2006), and neuroscientific perspectives (Youell, 2008). In this sense, playfulness is defined as an active tendency to be engaged in play or a personality trait that is stable and spontaneously appears in play (Wu et al., 2019), composed of elements such as spontaneity, active engagement, and sense of humor.

Previous research has found that fathers’ direct interaction (e.g., playing with children and reading to children) and indirect participation (e.g., developmental support, planning for the future) are both associated with the development of children’s playfulness (Lee and Kim, 2013). In turn, children’s playfulness can influence their peer competence (Hwang, 2006; Cho and Sung, 2020; Fung and Chung, 2023; Han and Yang, 2023). Additionally, according to the father-child relationship-activation theory (Paquette, 2004) and the broaden-and-build theory of positive emotions (Fredrickson, 2019), playfulness may serve as a valuable mediating variable between paternal involvement and children’s peer competence.

### 1.3 Chinese cultural context

The paternal role has historically been deeply rooted in Confucian cultural traditions in China, serving as a cornerstone of familial and societal order (Santos and Harrell, 2016). Traditional Chinese fatherhood emphasizes symbolic authority and emotional restraint, with fathers primarily fulfilling disciplinary and educational responsibilities (Li, 2020), while maintaining minimal involvement in daily childcare and play activities (Li and Lamb, 2016). In recent decades, however, sociocultural shifts driven by three interconnected factors have led to a rethinking of paternal responsibilities. First, theoretical advancement in positive development theory and father-child activation relationship theory, have highlighted the crucial role of paternal engagement in child development. Second, demographic policy shifts exemplified by the Three-Child Policy have intensified societal expectations for co-parenting (He and Zuo, 2023). Third, the proliferation of dual-earner households has rendered maternal monopoly over childcare both practically unsustainable and socially undesirable (Hong and Zhu, 2022). Recent empirical evidence indicates a notable shift in paternal behavior, with contemporary Chinese fathers exhibiting greater involvement in childcare and heightened emotional expressiveness relative to earlier generations (Liu et al., 2021). Nevertheless, structural barriers persist: quantitative analyses reveal persistent disparities in caregiving time allocation, while qualitative studies highlight enduring cultural resistance to redefining paternal roles beyond economic provision and discipline (Li, 2020; Wu et al., 2012). Nowadays, most countries in the world are overemphasizing academic work at the cost of providing time and space to play (Sahlberg and Doyle, 2019). Chinese traditional culture in particular, attaches great importance to children’s learning, while play is often equated with ‘wasting time’ (不务正业). Nevertheless, contemporary researchers and educators are increasingly acknowledging the developmental significance of play and playfulness in childhood. Notably, there are variations in defining playfulness based on geographical context within Chinese culture and Western culture. Western culture emphasis lies more on external characteristics such as self-expression and non-inhibition (Barnett, 2007), while Chinese culture focuses more on internal characteristics like deep engagement, self- persistence, and relaxation (Yu et al., 2003). Therefore, whether paternal involvement and children’s playfulness have different characteristics and how the variables effect young children’s peer competence within Chinese cultural background, needs to be further studied.

### 1.4 The present study

We aim to examine in the Chinese context: (1) The direct relationship between paternal involvement and young children’s peer competence. (2) The role of playfulness in the relationship. (3) Whether children’s playfulness can mediate the effect of paternal involvement on young children’s peer competence. We hypothesize that paternal involvement would be positively associated with young children’s peer competence and playfulness, and that young children’s playfulness would partially mediate the relationship between paternal involvement and young children’s peer competence.

## 2 Materials and methods

### 2.1 Participants and procedure

This study adopted the cluster random sampling method. Before distributing questionnaires, we randomly selected four kindergartens from four district in Yantai, Shandong Province, China. After obtaining the approval of kindergarten principals, during the family-kindergarten cooperation day, we first explain to the parents about the purposes and procedures of our study. Then, we guided fathers to report the paternal involvement questionnaire, and mothers to report the children’s playfulness and peer competence questionnaires. This study received approval from the Ethic Committee of the college of Education of Shandong Women’s University. And all participants read and signed the informed consent form. A total of 400 questionnaires were distributed and 41 were excluded, of which 29 fathers and 12 mothers did not fill out the questionnaire. The final analysis included 359 valid questionnaires, accounting for 89.75% of the total. The age of children ranged from 4 to 6 years old (*M* = 4.98, SD = 0.77), including 164 boys (45.7%) and 195 girls (54.3%), and 108 4 years old children (42.6%, 46 boys), 149 5 years old children (45.6%, 68 boys), 102 6 years old children (49.0%, 50 boys). Regarding education level, fathers were either high school graduates (26.5%) or had university/college degrees (62.4%). Households with a monthly income of more than 9,000 RMB accounted for 53.2%. The sample mainly consisted of participants from the middle class.

### 2.2 Tools

#### 2.2.1 Paternal involvement

Paternal involvement was assessed using the Chinese version of 26-item Father Involvement Questionnaire (FIQ) (Chen, 2021). The scale’s reliability and validity were rigorously examined, comprising three dimensions: Engagement (9 items, e.g., “accompanying child to a museum, zoo, science center, or library”), Accessibility (5 items, e.g., “when we are not together, my child can connect with me if he/she wants to”), and Responsibility (12 items, e.g., “providing financial support for child’s development”). Participants rated their responses on a scale ranging from 1 (never) to 5 (always), and item scores are averaged. Higher scores indicate greater paternal involvement. The Cronbach’s *α* coefficient of the total scale in the present study was 0.964.

#### 2.2.2 Playfulness

Children’s playfulness was assessed by the 23-item Children’s Playfulness Scale (CPS) (Barnett, 1990), which has been widely used in accessing playfulness of young children (e.g., Han and Yang, 2023; Fung and Chung, 2022). The scale consisted of five dimensions: Physical spontaneity (4 items, e.g., “the child is physically active during play”), Social Spontaneity (5 items, e.g., “the child initiates play with others”), Cognitive spontaneity (4 items, e.g., “the child uses unconventional objects in play”), manifest joy (5 items, e.g., “the child expresses enjoyment during play”), and sense of humor (4 items, e.g., “the child enjoys joking with other children”). CPS has been extensively utilized in Chinese research, and its reliability has been widely established. Participants rated their responses on a scale ranging from 1 (never) to 5 (always), and item scores were averaged. The Cronbach’s *α* coefficient of the total scale in the present study was 0.868.

#### 2.2.3 Peer competence

Peer competence was assessed using the Chinese version of 24-item children’s Ability to Associate with Partners Scale (AAPS) (Zhang, 2002), which has been widely used in China and its reliability and validity have been repeatedly confirmed. The scale consisted of four dimensions: Social Initiative (6 items, e.g., “the child actively introduce oneself to the new partner”), verbal and nonverbal ability to associate (6 items, e.g., “the child can use smiling, waving, nodding, and other gestures”), Social Dysfunction (6 items, e.g., “the child frequently experiences peer rejection”), and prosocial behavior (6 items, e.g., “the child demonstrates willingness to help other children”). Participants responded on a 5-point Likert scale ranging from 1 (never) to 5 (always), with item scores averaged for analysis purposes. The Cronbach’s α coefficient of the total scale in the present study was 0.851.

#### 2.2.4 Control variables

The children’s age and gender were assessed based on demographic information, and these variables were considered as covariates to ensure their effects were controlled.

### 2.3 Statistical analysis

The data were analyzed using SPSS 23.0 and Mplus 8.0 (Muthén and Muthén, 2017) in the present study. The data analysis comprised four steps.

Firstly, all variables were confirmed to meet normality assumptions, and Pearson correlation analyses were performed using SPSS 23.0. Secondly, confirmatory factor analysis of the scale was performed using Mplus 8.0, with x^2^/df < 3, TLI > 0.90, CFI > 0.90, and RMSEA <0.08 indicating a good fit (Little and Card, 2013). Thirdly, Latent variable structural equation modeling via Mplus 8.0 was employed to assess the mediating role of playfulness between paternal involvement and young children’s peer competence. Finally, the bootstrap method was utilized to obtain confidence intervals for testing both direct and indirect effects.

## 3 Results

### 3.1 Descriptive statistics and correlational analysis

Table 1 presents descriptive statistics and results of Pearson’s correlation analysis for each variable. The results showed that paternal involvement (*M* = 3.83, SD *=* 0.83), children’s playfulness (*M* = 4.02, SD = 0.52) and peer competence (*M* = 4.06, SD = 0.52), all exceeded the theoretical average level of 3.00, which represents the expected mean in the general population. Paternal involvement was positively correlated with playfulness (*r* = 0.45, *p* < 0.01) and young children’s peer competence (*r* = 0.31, *p* < 0.01). Children’s playfulness was positively correlated with their peer competence (*r* = 0.71, *p* < 0.01).

**Table 1:** Means, standard deviations, and inter-correlations among study variables (*N* = 359).

Variables	M(SD)	1	2	3
FI	3.82(0.83)	1		
PC	4.06(0.52)	0.45**	1	
PL	4.02(0.52)	0.31**	0.71**	1

FI, paternal involvement; PC, peer competence; PL, Playfulness. **p < 0.01 (two-tailed).

### 3.2 Mediation analyses

**Figure 1:**
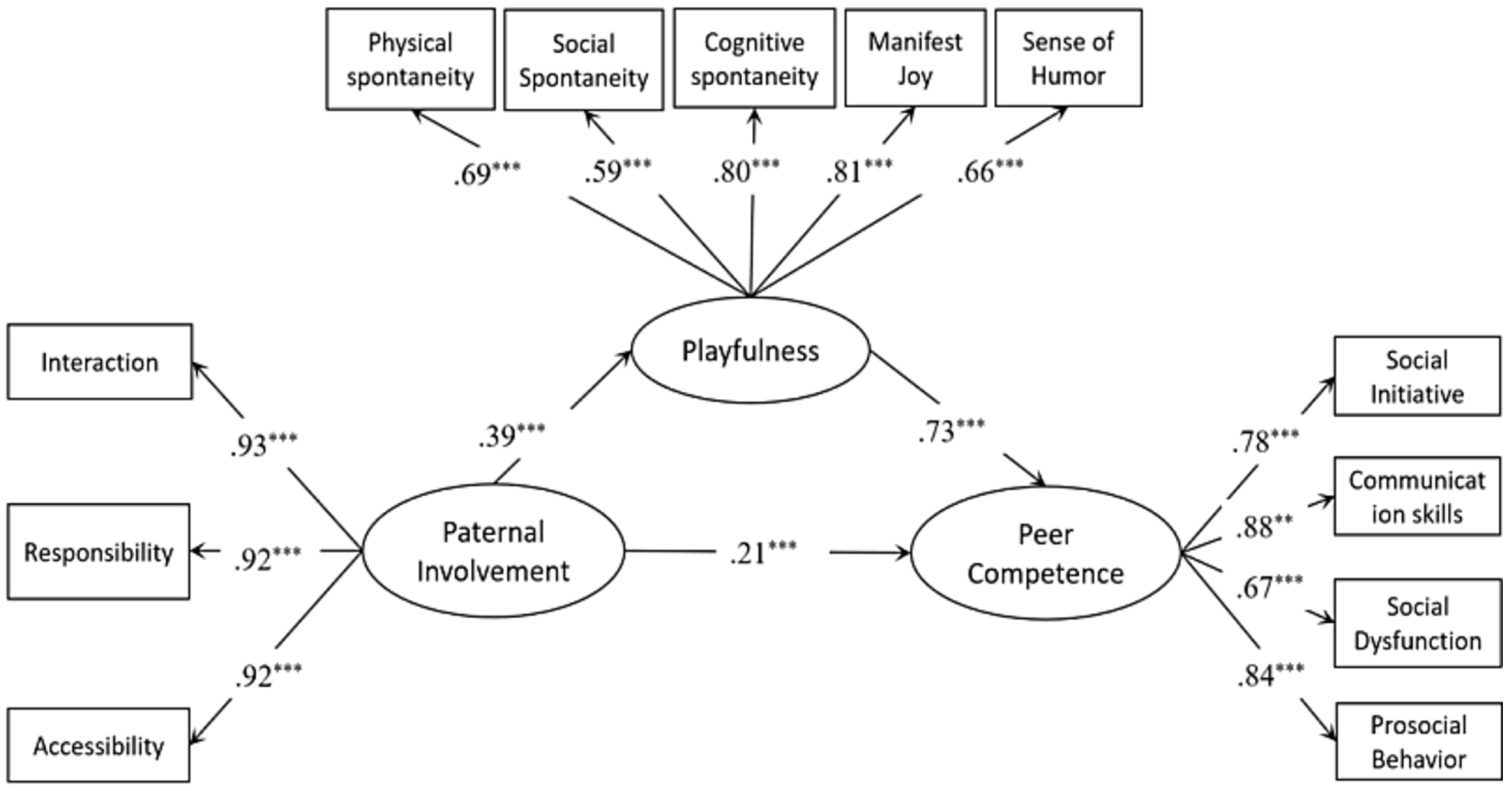
Mediating model of playfulness between father involvement and young children’s peer competence. RMSEA = 0.066, CFI = 0.94, TLI = 0.93 (*N* = 359). **p < 0.01, ***p < 0.001 (two-tailed).

The total effect model demonstrated an acceptable fit to the data (x^2^/df = 1.11, CFI = 1; TLI = 1; RMSEA = 0.034). As shown in Table 2, paternal involvement exhibited a significant total effect on children’s peer competence (*β* = 0.50, 95% CI: [0.39, 0.59]). Subsequently, we tested the mediation model, the mediation model displayed an acceptable fit to the data (x^2^/df = 3.18, *p* < 0.001; CFI = 0.94; TLI = 0.93; RMSEA = 0.066; SRMR = 0.058), and all the standardized path coefficients were presented in Figure 1. Specifically, although the direct effect attenuated yet still statistically significant (*β* = 0.21, 95% CI: [0.12, 0.32]), the positive predictive impact of paternal involvement on peer competence persisted in our findings and was partially mediated by children’s playfulness (*β* = 0.29, 95% CI: [0.20, 0.39]). Notably, approximately 58% of the total effect was accounted for by the mediation effect of children’s playfulness (as shown in Table 2).

**Table 2:** Standardized estimate and 95% CIs for direct and indirect effects.

Path effect	Path	Standardized estimate	95% confidence interval	
			Boot CI upper limit	Boot CI lower limit
Total effect	FI → PC	0.50^a^	0.39	0.60
Direct effect	FI → PC	0.21^a^	0.12	0.32
Indirect effect	FI → PL → PC	0.39 × 0.73 = 0.28^a^	0.20	0.39

a Empirical 95% confidence interval does not include zero.

FI, paternal involvement; PL, playfulness; PC, peer competence.

## 4 Discussion

The present study addressed an important issue: namely whether paternal involvement promotes young children’s peer competence in contemporary Chinese cultural contexts and what are the underlying mechanisms. We constructed a mediation model to investigate the impact of paternal involvement on young children’s peer competence and examine the mediating roles of children’s playfulness. The findings of our study hold theoretical implications and practical significance for facilitating paternal involvement, enhancing young children’s playfulness, and promoting their peer competence.

### 4.1 Paternal involvement predicts peer competence in young children

As expected, the relationship between paternal involvement and children’s peer competence is positive and significant. Which means, children whose fathers are positively involved in their lives tend to have better peer interaction skills. This finding aligns with previous studies (Amodia-Bidakowska et al., 2020; Craig et al., 2018; Liu et al., 2021; StGeorge and Freeman, 2017) and provides empirical support for attachment theory, parental capital theory, and father-child activation relationship theory. According to attachment theory, positive paternal involvement (e.g., warmth, sensitivity, supportiveness) can foster strong emotional bonds between fathers and children (Brown et al., 2018; Cabrera, 2020; Newland et al., 2008). These bonds enable children to access essential psychological resources, such as resilience and self-efficacy (Ladd and Parke, 2021), and help them develop positive internal working models, which can enhance their interpersonal trust and sense of security (Caina et al., 2016; Groh et al., 2014). This, in turn, may help them approach others with positive attitudes and expectations, ultimately improving their socialization skills. Parental capital theory also suggests that fathers can create opportunities for their children to engage with peers and offer advice on peer selection and appropriate play strategies, which is crucial in fostering early childhood social competence (Li et al., 2020).

The father-child activation relationship theory posits that direct interaction between fathers and children (e.g., rough and tumble play), often leads to heightened arousal levels. This can have a stimulating effect on children’s physical, emotional, and cognitive development while promoting self-control and empathy towards others (Paquette, 2004). A defining feature of father-child play involves numerous vigorous and playful physical interactions, which foster positive emotional experiences, including enjoyment, pleasure, and playfulness, for both fathers and children (Flanders et al., 2013). These shared experiences critically contribute to establishing and sustaining father-child emotional bonds (Paquette et al., 2021). The significance of emotional bonds in facilitating children’s social adaptation and interpersonal interactions has been corroborated by attachment theory and family-peer system linkage theory. In addition, research shows that high-quality father-child physical play incorporates unique parenting behaviors such as warmth, reciprocity, assertive control, sensitivity, touch, and playfulness (StGeorge and Freeman, 2017). This can creates a structured and meaningful environment that fosters confidence, cognitive and emotional control in children, as well as the ability to exercise restraint in competitive or conflictual situations (Paquette and StGeorge, 2023; Smith and StGeorge, 2023). Notably, children demonstrating advanced emotional regulation strategies and cognitive control mechanisms exhibit two critical developmental advantages: reduced externalizing problem and enhanced Social- emotional functioning (Lindsey and Berks, 2019; StGeorge et al., 2021; Wang and Feng, 2024). These made children more popular among peer groups and likely to emerge as leaders within them (Paquette, 2015).

### 4.2 Mediating role of playfulness

The present study also revealed a significant mediating role of playfulness in the relationship between paternal involvement and children’s peer competence. Specifically, in the first path of the mediator model, paternal involvement was significantly correlated with children’s playfulness. This finding aligns with previous studies (Lee and Kim, 2013) and provides empirical support for dynamic system theory and positive developmental theory. Lee and Kim (2013) revealed that paternal involvement (e.g., developmental support, child care and guidance) significantly accounted for the variance in the playfulness of young children. Some studies have reported that paternal support and positive responsiveness during early childhood are predictors of children’s cognitive and socio-emotional skills (Cabrera et al., 2007). Moreover, cognitive and emotional skills have been proven to influence children’s playfulness (Jo et al., 2016). According to dynamic system theory and positive developmental theory, development occurs in an open system of self-organization, characterized by a continuous exchange of information with the environment. Furthermore, most developmental phenomena are influenced by multiple factors and exhibit temporal changes (Fischer and Bidell, 2006). Playfulness occurs during early experiences of playful interaction with parents (Reddy, 1991; Youell, 2008), through which children develop a cognitive perception of the world as a playground and perceive the external environment as friendly and secure (Paquette, 2004). Consequently, they display increased engagement, exploratory behavior, and positive emotion during play activities with peers. This playful state recurs in parent–child and child–child interactions, gradually internalizing into individuals’ behavior patterns.

In the second path of the mediator model, children’s playfulness showed a significant association with their peer competence. Playful children typically exhibit high levels of cognitive, social and physical spontaneity (Lieberman, 1965; Singer and Rurnmo, 1973). This enables them to better plan and execute play actions, generate attractive play ideas, and effectively engage play partners (Fung and Chung, 2022). Additionally, they are proficient in using conflict resolution strategies such as compromise, cooperation, avoidance, and concession when interacting with peers (Hwang, 2006; StGeorge and Freeman, 2017). Moreover, children with playfulness have greater cognitive flexibility and are more resourceful in establishing positive peer play interactions. They can more naturally enter into peer play, negotiate roles and directions, and resolve conflicts constructively (Fung and Cheng, 2017; Fung and Chung, 2023). These playful qualities make children more popular with their peers. Conversely, children lacking in playfulness often exhibit social withdrawal, which hinders the display of prosocial behavior and increases the likelihood of peer rejection or exclusion (Han and Yang, 2023; Kim and Kim, 2019; Rubin et al., 2018).

Furthermore, in line with Fredrickson’s broaden-and-build theory of positive emotions, playfulness is regarded as a psychological or emotional state in which children actively engage in play and derive pleasure. It plays a pivotal role in the expansion and constructive effect on children’s social development. On one hand, playfulness has a protective expansion effect against individual negative characteristics, such as shyness and social indifference, shielding children from peer play rejection or isolation (Han and Yang, 2023). On the other hand, it has a construction function of expanding positive individual characteristics, such as joy, interest, and a sense of humor and fulfillment (Wu et al., 2019). Children with positive emotions are more likely to evoke positive emotional expressions and adaptive coping strategies (Cho and Sung, 2020; Wang and Feng, 2024).

The findings suggest that positive and affectionate father-child interactions are crucial for the development of children’s peer competence. The study emphasizes the importance of child-centered and play-based activities in the home environment to foster children’s playfulness and social development.

## 5 Contributions and limitations

### 5.1 Contributions

There are some contributions to this study: (1) In this study, we expanded upon previous investigations into the mechanisms underlying the impact of paternal involvement on young children’s peer competence in light of the dynamic system theory, social cognitive theory, father-child relationship-activation theory and broaden-and-build theory of positive emotions. The findings revealed a positive correlation between higher levels of paternal involvement and enhanced peer competence among young children in contemporary Chinese society. Furthermore, our results demonstrated that children’s playfulness played a significant partial mediating role in the association between paternal involvement and young children’s peer competence, highlighting the crucial importance of positive personality traits for family interactions and child development. (2) From a practical perspective, our study underscores the significance of child-centered and play-based activities within the home environment for promoting children’s peer competence. Additionally, we emphasizes the value of fathers’ positive life guidance and affectionate behavior during interactions with their children during playtime. Our study offers targeted guidance and theoretical support for facilitating positive paternal involvement that effectively enhances children’s playfulness and peer competence. Consequently, governmental implementation of fertility support policies is warranted to ensure sufficient father-child bonding time while communities and kindergartens should enhance fathers’ awareness regarding parenting roles through family education lectures or guidance sessions to ensure high-quality paternal involvement. (3) Father and mother exhibit overlapping roles yet demonstrate distinct contributions in the socio-emotional development of children. Specifically, the emotional scaffolding provided by mothers and the social problem-solving modeling exhibited by fathers constitute complementary yet independent developmental mechanisms. Consequently, family systems should strategically encourage active paternal involvement in caregiving practices, thereby establishing a co-parenting framework that optimizes developmental outcomes through the integration of dual parental resources.

### 5.2 Limitations

Future researches should address some limitations to this study: (1) Utilized a small sample size selected from four public kindergartens in one specific city in eastern China, which may limit the generalizability of the findings. Future research should consider examining paternal involvement across various significant regional and ethnic differences observed in different provinces throughout China, as well as sampling from both urban and rural settings and different types of kindergartens. (2) While our study exclusively employed a questionnaire-based methodology, Subsequent investigations should consider adopting multi-method approaches such as direct observational measures of paternal-child interactions, peer play dynamics, and tests of social competence. Such methodological triangulation would enable more rigorous evaluation of whether parent-reported correlations authentically reflect children’s behavioral manifestations. (3) The present study solely focused on exploring the role of paternal factor on young children’s peer competence, however, development is influenced by multiple systems. Therefore, future research should investigate how family dynamics, school environments, and individual variables systematically influence young children’s peer competence.

## Data Availability

The raw data supporting the conclusions of this article will be made available by the authors, without undue reservation.
