# Correction: Paternal involvement and peer competence in young children: the mediating role of playfulness

**DOI:** 10.3389/fpsyg.2025.1731169

**Published:** 2025-11-03

**Authors:** Chunyan Liang, Xinwen Bi

**Affiliations:** Department of Education, Shandong Women's University, Jinan, Shandong Province, China

**Keywords:** paternal involvement, young children, playfulness, peer competence, Chinese context

An incorrect **Funding** statement was provided. The correct funder is High-Level Talents of Shandong Women's University. The correct **Funding** statement reads:

The author(s) declare that financial support was received for the research and/or publication of this article. This study was supported by the Scientific Research Fund for High-Level Talents of Shandong Women's University (2020RCYJ08).

The original version of this article has been updated.

